# Heart rate sensitivity of virtual non-contrast calcium scores derived from photon counting detector CT data: a phantom study

**DOI:** 10.1007/s11547-024-01773-3

**Published:** 2024-02-06

**Authors:** Franka Risch, Florian Schwarz, Thomas Kroencke, Josua A. Decker

**Affiliations:** 1 Department of Diagnostic and Interventional Radiology, University Hospital Augsburg, Stenglinstr. 2, 86156 Augsburg, Germany; 2 Medical Faculty, Ludwig Maximilian University Munich, Munich, Germany; 3 Centre for Advanced Analytics and Predictive Sciences (CAAPS), University Augsburg, Augsburg, Germany; 4 Clinic for Diagnostic and Interventional Radiology, Donau-Isar-Klinikum, Deggendorf, Germany

**Keywords:** Photon-counting detector computed tomography, Heart rate susceptibility, Virtual non-contrast imaging, Calcium scoring

## Abstract

**Purpose:**

To assess the reliability of virtual non-contrast (VNC) derived coronary artery calcium quantities in relation to heart rate and the VNC algorithm used compared to reference true non-contrast (TNC), considering several clinically established acquisition modes.

**Material and methods:**

An ad hoc built coronary phantom containing four calcified lesions and an iodinated lumen was scanned using three cardiac acquisition modes three times within an anthropomorphic cardiac motion phantom simulating different heart rates (0, 60, 80, 100 bpm) and reconstructed with a conventional (VNC_conv_) and a calcium-sensitive (VNC_pc_) VNC algorithm. TNC reference was scanned at 0 bpm with non-iodinated lumen. Calcium scores were assessed in terms of number of lesions detected, Agatston and volume scores and global noise was measured. Paired t-test and Wilcoxon test were performed to test measurements for significant difference.

**Results:**

For both VNC algorithms used, calcium levels or noise were not significantly affected by heart rate. Measurements on VNC_pc_ reconstructions best reproduced TNC results, but with increased variability (Agatston scores at 0 bpm for TNC, VNC_conv_, and VNC_pc_ were 47.1 ± 1.1, 6.7 ± 2.8 (*p* < 0.001), and 45.3 ± 7.6 (*p* > 0.05), respectively). VNC reconstructions showed lower noise levels compared to TNC, especially for VNC_pc_ (noise_heart_ on TNC, VNC_conv_ and VNC_pc_ at 0 bpm was 5.0 ± 0.4, 4.5 ± 0.2, 4.2 ± 0.2).

**Conclusion:**

No significant heart rate dependence of VNC-based calcium scores was observed in an intra-reconstruction comparison. VNC_pc_ reproduces TNC scores better than VNC_conv_ without significant differences and decreased noise, however, with an increasing average deviation with rising heart rates. VNC-based CACS should be used with caution as the measures show higher variability compared to reference TNC and therefore hold the potential of incorrect risk categorization.

**Supplementary Information:**

The online version contains supplementary material available at 10.1007/s11547-024-01773-3.

## Introduction

The appearance and extent of coronary artery calcium (CAC) is a reliable indicator for coronary artery disease and coronary atherosclerosis, and an established predictor of cardiovascular risk [1, 2]. By means of the radiopacity of calcified plaques, computed tomography (CT) can provide a fast and non-invasive evaluation of CAC [3]. Usually, the extent of calcium in coronary arteries is quantified on non-enhanced CT scans, followed by an angiography for stenosis evaluation [4]. Spectral CT information, provided by dual-energy or photon-counting detector CT (PCD-CT) acquisitions, allow post-processing steps including the virtual removal of the iodinated contrast medium resulting in virtual non-contrast (VNC) images [5]. Many studies investigated the possibility of calcium scoring on VNC reconstructions and found excellent correlations which promise to reduce the radiation exposure to solely the CT angiography and omitting an additional unenhanced scan [6–9]. A relevant challenge in cardiac imaging is motion which might lead to artifacts and corresponding misinterpretation of the CAC. Werf et al. [10] found in their multi-manufacturer system phantom study a significant influence of heart rate on measured calcium quantities. To transfer such analyses on VNC reconstructions, there is a limited availability of suitable coronary phantoms. So far, phantoms either provide calcified plaques in combination with blood equivalent lumen, the focus is on stenosis analysis and non-calcified plaques are simulated, or comparison to reference true non-contrast (TNC) is lacking [10–13].

For this study, a coronary vessel phantom including calcifications embedded in iodinated agarose was built to fit within an anthropomorphic cardiac motion phantom. Different heart rates were simulated and clinical coronary angiography CT scans were performed on a photon-counting detector system. Calcium scores were measured on virtual non-contrast reconstructions and compared to TNC reconstructions.

## Materials and methods

Because the study uses only phantom data, an ethics approval was not required.

### Phantom

The phantom setup consists of two parts: The dynamic cardiac phantom and the coronary vessel phantom.

Former is an anthropomorphic heart inside a thorax body from tissue equivalent materials (MODEL 008C, Computerized Imaging Reference Systems Inc., Virginia, USA). A cylindric part containing the heart can be controlled to perform motions of variable heart rate combining translation and rotation with the possibility to read out the correlating electrocardiographic profile. Three 5 mm-diameter accessible cutouts in the heart simulate the left coronary artery and can be individually filled with inserts.

In the absence of commercially available inserts simulating coronary arteries with calcified plaques and the ability to alternate the lumen, a suitable phantom was developed. A rigid plastic tube with an outer diameter of 5 mm was cut lengthwise and pieces mimicking common sizes of coronary calcified plaques, ranging from 0.3 mm to 0.7 mm as the longest diameter of the same calcium tablet (2028.9 mg calcium carbonate each of two tablets and modified starch, Vitamaze GmbH, Heidelberg, Germany) were placed and glued inside. Agarose powder (1.4 g for 100 ml solution) was dissolved in a mixture of iodine (Ultravist 300, Iopromide, Bayer Vital GmbH, Leverkusen, Germany) and sodium chloride (0.9%) (ratio 1/20). The plastic tube was embedded in the solution and cooled in a refrigerator to gel. The surrounding agarose was removed, and the filled plastic tube was placed in a heat-shrinkable tube and sealed on both sides. For reference TNC scans, the shrink tube was removed and the iodinated agar was washed out. All steps of embedding the tube were repeated without adding iodine.

**Table 1:** Phantom and image acquisition and reconstruction settings

Phantom	With iodine	Without iodine
Heart rate (bpm)	0/ 60/ 80/ 100	0
Scan mode	Flash/ spiral/ sequence	Flash/ spiral/ sequence
Tube voltage (kVp)	120	120
Repetitions	3	3
Kernel, iteration	Qr36, Q3	Qr36, Q3
Slice thickness, increment (mm)	3.0, 1.5	3.0, 1.5
Post-processing	VNC_conv_/ VNC_pc_ 65 keV	VMI 65 keV (= TNC)

Qr = quantitative regular kernel, VMI = virtual monoenergetic imaging, VNC = virtual non-contrast (conv = conventional, pc = PureCalcium)

### CT protocol

The phantom including the iodinated vessel was scanned at heart rates of 0, 60, 80, and 100 bpm. Without iodine the phantom was only scanned at 0 bpm as reference. All scans were performed on a PCD-CT system (NAEOTOM Alpha, Siemens Healthcare GmbH, Erlangen, Germany) in December 2022. All three available scan modes for cardiac imaging, flash (corresponds to high-pitch spiral), spiral, and sequence were used at a tube voltage of 120 kVp with a constant image quality level of 70 to adjust the tube current–time product and repeated three times. The spiral and sequence acquisition modes were electrocardiographically triggered, using the best diastole. An acquisition mode with spectral information readout (Quantum Plus, Siemens Healthcare GmbH, Erlangen, Germany) was used and the collimation was 144 × 0.4 mm. Details of the settings for image acquisition and reconstruction are provided in Table 1.

### Image reconstruction

All scans were reconstructed at the scanner console (syngo, VA50, Siemens Healthcare GmbH, Erlangen, Germany) using the quantitative regular kernel Qr36 with an iteration strength of three. Slice thickness and increment were 3.0 mm and 1.5 mm, respectively, and the field of view 180 × 180, covering the heart. CT data of the iodinated vessel phantom were processed using two virtual non-contrast algorithms, the conventional VNC_conv_ and the calcium-preserving VNC_pc_. TNC scans of the phantom without iodine were reconstructed at a virtual monoenergetic level of 65 keV.

### Image analyses

Calcium quantities, number of recognized lesions (CAC_Number_), volume (CAC_Volume_) and Agatston (CAC_Agatston_) score, were acquired semi-manually on a dedicated workstation (syngo.via, version VB60A, Siemens Healthcare GmbH, Erlangen, Germany), considering contiguous voxels with an attenuation above a threshold of 130 HUs. Measurements were taken for each reconstructed series and exported in tabular form.

For noise analysis, volumes were cropped to 50 slices in axial direction to cover only the heart. Three slices, approximately equidistant from each other and from the range boundaries (slice 13, 26, 39) (see supplemental Fig. 1a), were selected and their noise map calculated. Following Christianson et al. [14], a filter of 6 mm size, referring to 17 pixels (512 pixel in rows and columns with a FoV of 180 result in a pixel size of 0.352 mm) was used to calculate the standard deviation of CT-values for each image pixel (see supplemental Fig. 1b). A rectangular region of interest (ROI) covering the moving cylinder (the heart) of the phantom was used to distinguish between the dynamic heart and the static background. Histograms of the noise map allow the detection of the most frequent occurring standard deviation within one axial slice, which was used as a measure for global noise, in both regions, heart and background, respectively (see supplemental Fig. 1c). The global noise values were averaged over the three slices considered, resulting in two global noise values, for heart (noise_heart_) and background (noise_background_), for each reconstruction.

**Fig. 1:**
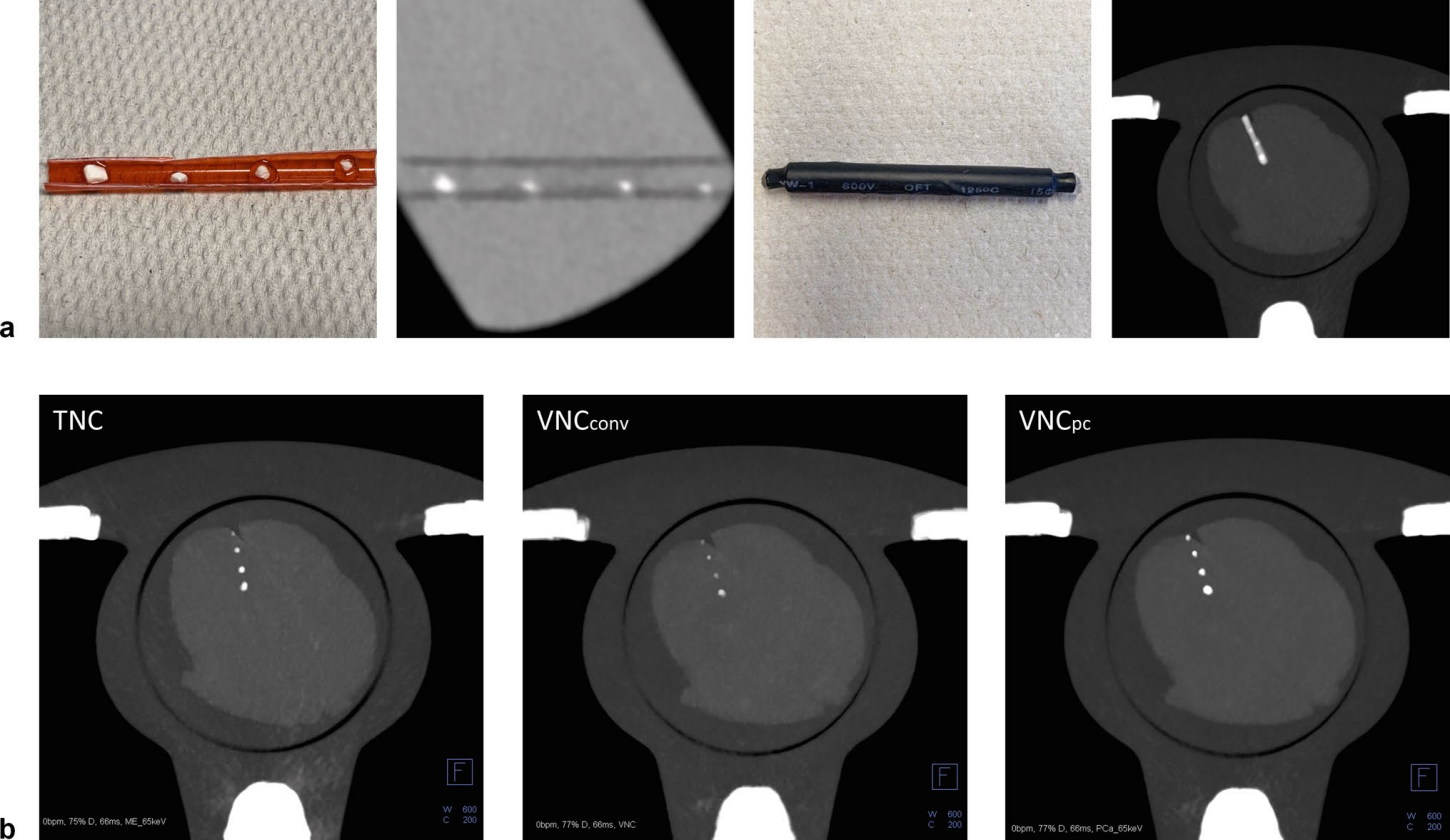
**a** shows the development of the coronary vessel phantom from gluing the pieces of CaCO_3_ into the tube, to embedding the phantom in iodinated agarose, to the final phantom covered and sealed in a heat-shrinkable tube inside the cardiac phantom. **b** shows a maximum intensity projection of the reconstruction modes for the true non-contrast (TNC), the conventional and PureCalcium virtual non-contrast reconstruction (VNC_conv_ and VNC_pc_) at equal heart rate, scan and reconstruction settings

### Statistical analyses

Statistical analyses were performed using Python (version 3.9). The Shapiro–Wilk test was used to assess the distribution of the data. The paired t-test and the Wilcoxon signed-rank test were used to test for differences, for parametric and nonparametric data, respectively. For multiple comparisons, *p*-values were corrected using the Bonferroni method and considered to indicate statistical significance if < 0.05. CAC and noise were once compared between different heart rates within each reconstruction and once between different reconstructions within each heart rate, visualized in box plots. Percentage deviations were calculated as $\frac{{X}_{{\text{VNC}}}-{X}_{{\text{TNC}}}}{{X}_{{\text{TNC}}}}*100\mathrm{ \%}$, with *X*_VNC_ representing CAC or noise derived from a VNC series (either conventional or PureCalcium) and *X*_TNC_ derived from ground truth. Differences due to scan modes (flash, spiral, sequence) were not evaluated. However, each mode is used in clinical practice and therefore represented in equal proportions in this study (see CT protocol and image reconstruction section).

## Results

### Coronary phantom

Figure 1a shows the different stages of development of the coronary phantom insert. Embedded in iodinated agar, as shown in the second image, the mean measured CT values of the calcifications ranged from 790 ± 50 HU for the smallest to 1120 ± 110 HU for the largest volume. The contrast within the tube was measured to be 500 ± 25 HU. Figure 1b shows the reconstructions of TNC and VNC of the cardiac motion phantom including the coronary insert are shown as maximum intensity projections at 0 bpm and the same scan and reconstruction settings. The four placed calcifications are clearly visible in all reconstructions.

### Dose

Pitch and dose parameters, including volumetric dose index (CTDI_vol_), dose length product (DLP), and size-specific dose estimate (SSDE), are listed in Table 2. Since the acquisition settings were kept identical, equivalent doses were used for TNC and CTA scans. Small deviations are due to variations in the manual selection of the scan area.

**Table 2:** Measured dose parameters for the coronary vessel scanning with and without iodinated lumen, including three repetitions and three scan modes per heart rate

Phantom	With iodine				Without iodine
Heart rates	0 bpm	60 bpm	80 bpm	100 bpm	0 bpm
Pitch factor	0.8 (0.2–3.2)	0.8 (0.2–3.2)	0.8 (0.3–3.2)	0.8 (0.3–3.2)	0.8 (0.2–3.2)
Eff. mAs	38	38	38	38	38
CTDI_vol_ (mGy)	5.9 (2.7–13.7)	6.2 (2.8–43.8)	6.2 (2.7–33.7)	6.1 (2.7–27.4)	6.1 (2.8–43.8)
DLP (mGy*cm)	55.8 (47.8–176.0)	86.9 (48.0–480.0)	86.9 (47.9–507.0)	86.5 (47.9–415.0)	86.6 (61.3–652.0)
SSDE (mGy)	9.7 (4.4–24.3)	10.1 (4.4–70.1)	10.1 (4.4–53.9)	10.1 (4.4–43.8)	10.1 (4.5–69.9)

Values are median (interquartile range). CTDI_vol_ = volumetric CT dose index, DLP = dose length product, SSDE = size-specific dose estimate

### Calcium scoring

**Fig. 2:**
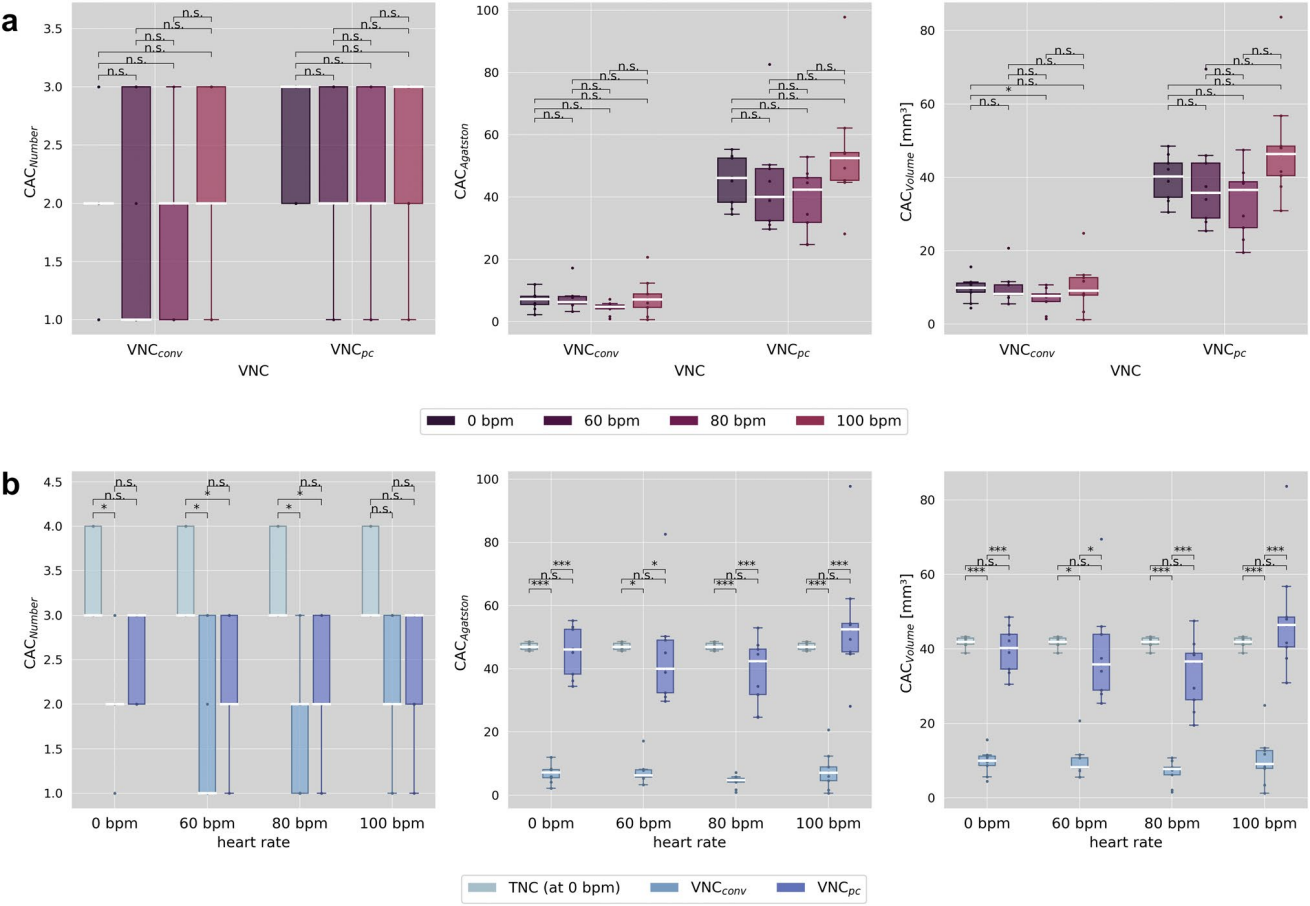
shows the measured number of calcified lesions (CAC_Number_), the Agatston score (CAC_Agatston_) and the volume (CAC_Volume_) in **a** comparing the heart rates for the respective reconstructions of conventional and PureCalcium virtual non-contrast (VNC_conv_ and VNC_pc_) and in **b** comparing the reconstructions TNC at 0 bpm versus VNC at heart rates 0, 60, 80 and 100 bpm

Detailed CAC measurements, including the percentage differences from TNC results, are shown in Table 3. There is a trend for both VNC algorithms, yet more pronounced for VNC_pc_, to have similar CAC measurements at 60 bpm, lower at 80 bpm, and higher at 100 bpm compared to each algorithm's measurement at 0 bpm. However, as shown in Fig. 2a, the difference caused by heart rate is not significant for either the CAC measurement or the VNC algorithm (almost all p's > 0.05, *p*-value for VNC_conv_ 0 vs. 80 bpm = 0.047).

**Table 3:** Measured calcified lesions in number, Agatston and volume score at heart rates of 0, 60, 80, 100 bpm in true non-contrast and virtual non-contrast, conventional and PureCalcium, series

Heart rate (bpm)	Recon	Coronary artery calcification			Percentage difference to TNC (%)		
		Number	Agatston	Volume (mm^3^)	Number	Agatston	Volume (mm^3^)
0	TNC	3 (3–4)	47.1 ± 1.1	41.8 ± 1.3			
	VNC_conv_	2 (2–2)	6.7 ± 2.8	9.7 ± 3.3	− 40 ± 24	− 86 ± 6	− 77 ± 8
	VNC_pc_	3 (2–3)	45.3 ± 7.6	39.9 ± 6.1	− 21 ± 18	− 4 ± 17	− 4 ± 16
60	VNC_conv_	1 (1–3)	7.5 ± 4.0	10.1 ± 4.4	− 49 ± 26	− 84 ± 8	− 76 ± 10
	VNC_pc_	2 (2–3)	44.4 ± 16.2	38.8 ± 13.5	− 35 ± 19	− 6 ± 34	− 8 ± 32
80	VNC_conv_	2 (1–2)	4.4 ± 2.0	6.8 ± 3.2	− 46 ± 24	− 91 ± 4	− 84 ± 8
	VNC_pc_	2 (2–3)	38.8 ± 10.2	33.4 ± 9.3	− 38 ± 23	− 17 ± 22	− 20 ± 24
100	VNC_conv_	2 (2–3)	7.7 ± 6.1	10.3 ± 6.8	− 37 ± 26	− 84 ± 13	− 75 ± 16
	VNC_pc_	3 (2–3)	54.3 ± 18.8	48.2 ± 15.2	− 27 ± 25	15 ± 38	15 ± 35

Values are median (interquartile range) or mean ± standard deviation. TNC = true non-contrast, VNC = virtual non-contrast, conv = conventional, pc = PureCalcium

Focusing on the differences between the reconstructions, as in Fig. 2b, only TNC at 0 bpm was able to detect all four calcium lesions (median of 3 (3–4) lesions). With VNC_conv_, for most of the heart rates, there were two lesions with a median of more than 130 HU that could be counted, and only one lesion at 60 bpm. In general, more lesions were found on VNC_pc_ reconstructions (e.g. at 0 bpm 2 (2–2) on VNC_conv_ and 3 (2–3) on VNC_pc_) and the percentage difference to TNC was smaller compared to the conventional VNC algorithm (e.g. at 0 bpm − 40% for VNC_conv_ and − 21% for VNC_pc_), however, differences between VNC_conv_ and VNC_pc_ were not significant. At least one calcification was detected at all heart rates.

**Fig. 3:**
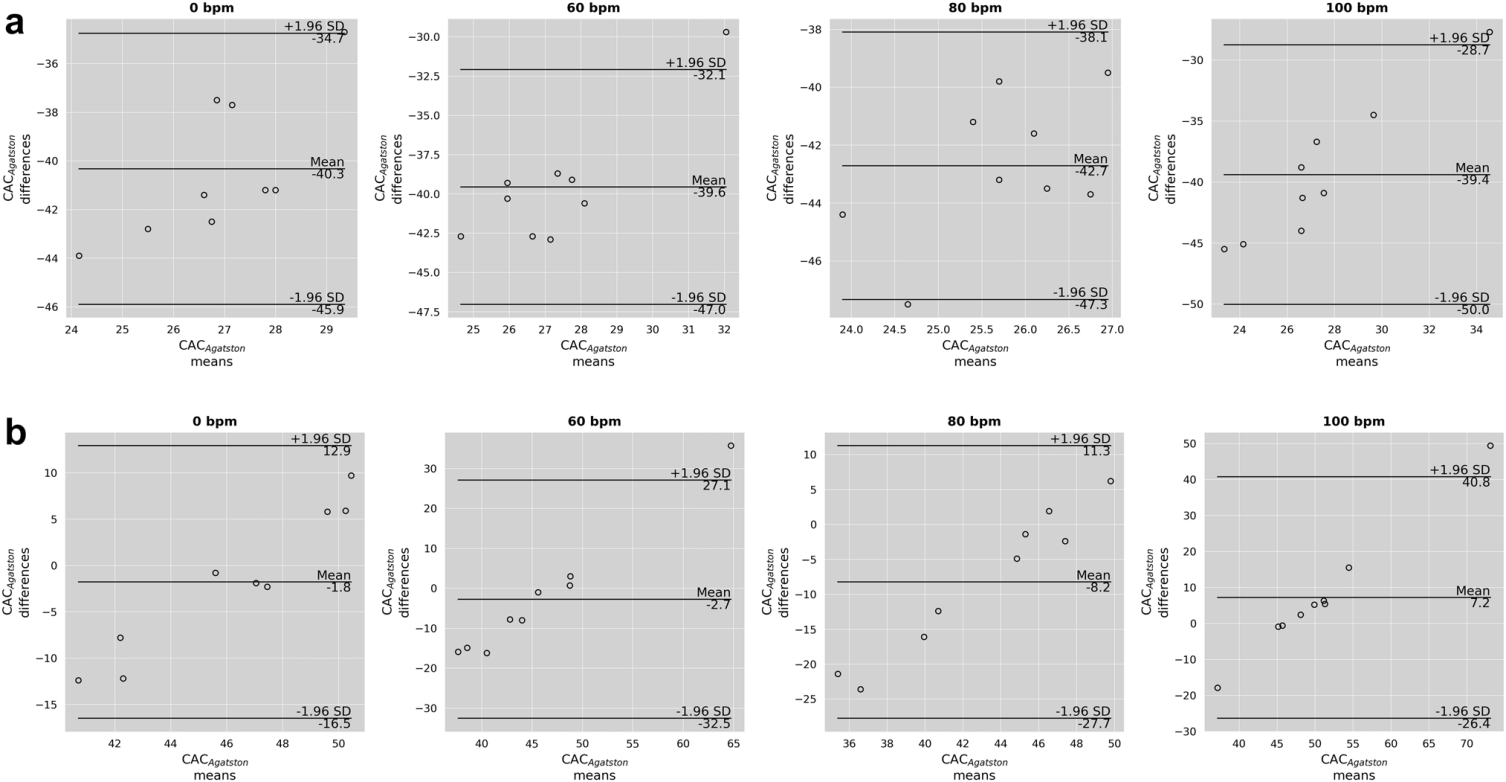
Bland Altman plots showing the means and differences of the Agatston scores measured in **a** conventional virtual non-contrast (VNC_conv_) and **b** PureCalcium virtual non-contrast (VNC_pc_) at 0, 60, 80 and 100 bpm versus measured in true non-contrast at 0 bpm

The VNC_pc_ derived Agatston scores did not differ significantly from the ground truth (TNC at 0 bpm), but the interquartile range and standard deviation were greater (e.g. at 0 bpm Agatston score on TNC of 47 ± 1 and on VNC_conv_ of 45 ± 8). The percentage difference was smallest at 0 bpm with an underestimation of TNC Agatston scores about − 4%, increasing to -17% at 80 bpm. At 100 bpm Agatston scores were overestimated by 15%. For VNC_conv_, the highest underestimation of scores was observed for 80 bpm with—91%. For the heart rates 0, 60 and 100 bpm, the underestimations was about—85%. The Bland–Altman plot in Fig. 3 shows the difference VNC—TNC over the respective means. It is noticeable that for VNC_conv_ (Fig. 3a) the distribution appears unstructured, resulting in a constant bias across heart rates. However, for VNC_pc_ (Fig. 3b) the distribution appears linear, with smaller differences at lower means and larger differences at higher means.

Similar results are found for CAC volume score measurements. Interestingly, compared to corresponding Agatston scores the percentage difference to TNC is smaller for VNC_conv_ (percentage difference to groundtruth at 80 bpm: Agatston score − 91%, volume score − 84%) and greater for VNC_pc_ (Agatston score − 17%, volume score − 20%). It should to be noted that although the variability seems to be more pronounced for VNC_pc_ measurements compared to VNC_conv_ ones, a transformation based on linear correlation includes an intercept and a slope and would naturally also increase the range, especially regarding the outliers. However, a valid observation is that at 0 bpm heart rate, TNC showed the least variability compared to VNC derived CACs.

### Noise

**Fig. 4:**
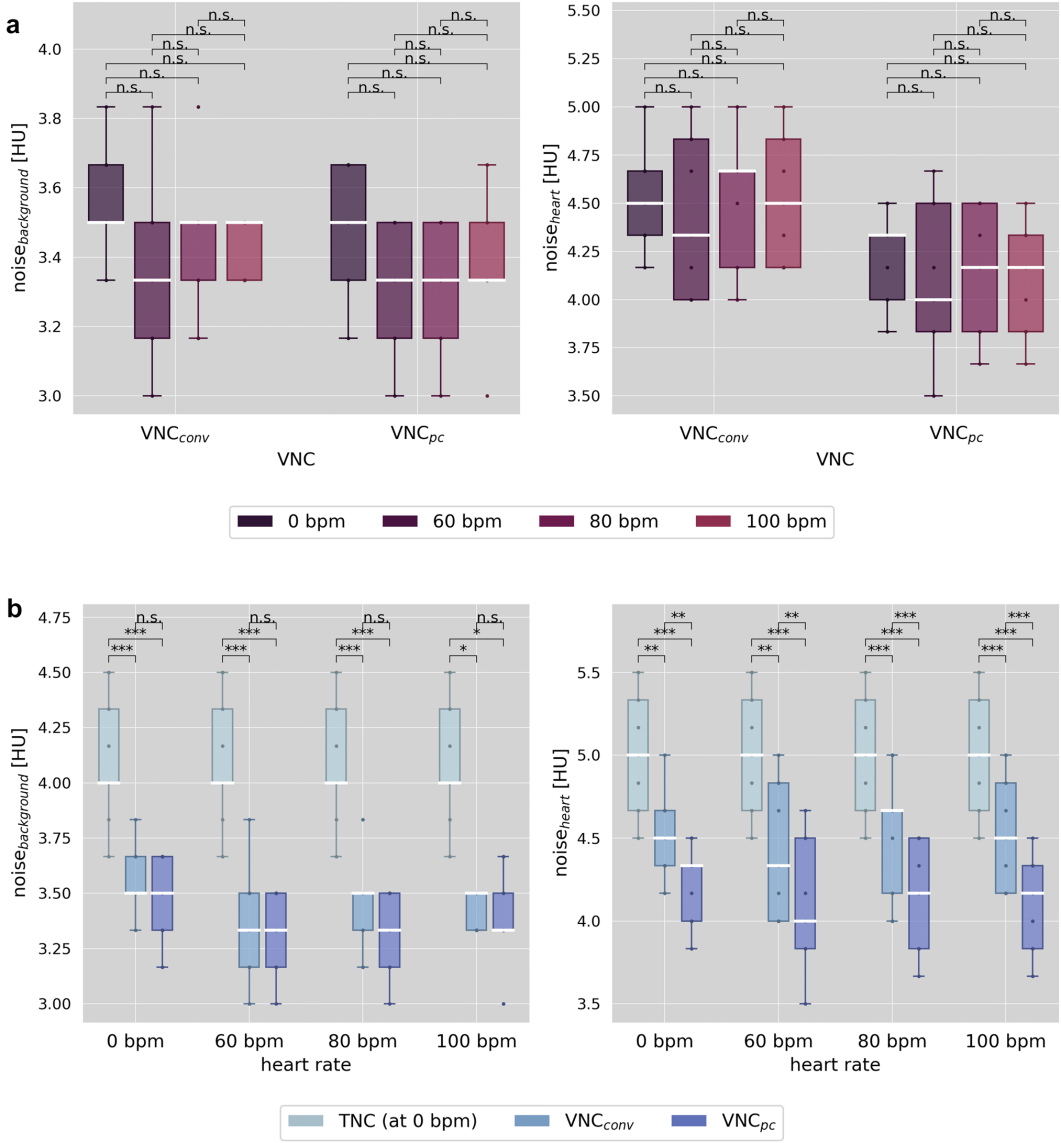
Depicts the measured global noise as the most frequent standard deviation in HU over the heart and the background in **a** comparing the heart rates within the respective reconstructions of conventional and PureCalcium virtual non-contrast (VNC_conv_ and VNC_pc_) and **b** comparing the reconstructions TNC at 0 bpm versus VNC at heart rates 0, 60, 80 and 100 bpm

Detailed global noise level measurements, including the percentage differences from TNC results, are shown in Table 4. In Fig. 4a, the heart rates within each VNC algorithm are the focus of comparison. Neither for VNC_conv_ nor for VNC_pc_ did the heart rates cause significant differences in noise levels (all *p*-values > 0.05). Focusing on the different reconstruction methods, as shown in Fig. 4b, TNC at 0 bpm has the highest noise level (5.0 ± 0.4 HU and 4.1 ± 0.3 HU in heart and background), which differs significantly from both VNC reconstructions for all heart rates and ROI regions (*p*-values < 0.05 or < 0.001). Within the static background, VNC_conv_ showed a lower noise level compared to TNC with a reduction from a minimum of 13% at 0 bpm to a maximum of 18% at 60 bpm. For VNC_pc_ the reduction was slightly higher, ranging from 15 to 19%. However, there was no significant difference in noise between the VNC algorithms. Noise in the dynamic heart region generally exceeded background measurements by approximately 1 HU. Although here the VNC algorithms barely differed in absolute global noise level (approximately 0.3 HU difference, but all p-values < 0.05) the percentage reduction compared to TNC was higher for VNC_pc_ with up to 18% less noise than for VNC_conv_ with a maximum of 12% less noise.

**Table 4:** Measured global noise levels for heart and background at heart rates of 0, 60, 80, 100 bpm in true non-contrast and virtual non-contrast, conventional and PureCalcium, series

Heart rate (bpm)	Recon	Global noise level (HU)		Percentage difference to TNC (%)	
		Heart	Background	Heart	Background
0	TNC	5.0 ± 0.4	4.1 ± 0.3		
	VNC_conv_	4.5 ± 0.2	3.6 ± 0.1	− 9 ± 6	− 13 ± 4
	VNC_pc_	4.2 ± 0.2	3.5 ± 0.2	− 16 ± 3	− 15 ± 7
60	VNC_conv_	4.4 ± 0.4	3.4 ± 0.3	− 12 ± 6	− 8 ± 5
	VNC_pc_	4.1 ± 0.4	3.3 ± 0.2	− 18 ± 7	− 19 ± 5
80	VNC_conv_	4.5 ± 0.4	3.4 ± 0.2	− 10 ± 4	− 17 ± 3
	VNC_pc_	4.2 ± 0.3	3.3 ± 0.2	− 17 ± 3	− 19 ± 3
100	VNC_conv_	4.5 ± 0.4	3.4 ± 0.1	− 10 ± 4	− 16 ± 4
	VNC_pc_	4.1 ± 0.3	3.5 ± 0.2	− 18 ± 5	− 17 ± 2

Values are mean ± standard deviation. TNC = true non-contrast, VNC = virtual non-contrast, conv = conventional, pc = PureCalcium

## Discussion

For this study, we scanned an ad hoc coronary vessel in a cardiac motion phantom at various heart rates using standard cardiac protocols. Calcification and image noise were quantified on VNC images derived from the dynamic contrast-enhanced phantom scans and on TNC reference, reconstructed from static unenhanced scans. The focus of the evaluation was on the heart rate sensitivity of the two used VNC reconstruction algorithms, and on their performance against each other and against the reference TNC. Our main important findings are: (1) VNC_pc_ reproduces TNC better than VNC_conv_ in terms of found calcium lesions, Agatston and volume score, with predominantly no significant differences; (2) The average discrepancy, however, increases with higher heart rates; (3) A high variability of measured calcium quantities on VNC reconstructions indicates a poorer reproducibility and dependence on the scan modes used compared to TNC, which carries the potential for incorrect risk classification; (4) Noise is reduced in VNC reconstructions compared to TNC, equally for the static background and more pronounced for VNC_pc_ within the dynamic cardiac region.

VNC reconstructions, especially in combination with the calcium-preserving algorithm (VNC_pc_), have proven their clinical relevance as a replacement for TNC in several applications. Recent studies have investigated, for example, the evaluation of patients after EVAR [15], quantification of epicardial adipose tissue [16] or the quantification of coronary artery calcium [8, 17, 18] based on VNC_pc_ reconstructions with promising results. Regarding the latter, the calcium sensitivity of VNC_pc_’s provides TNC-equivalent values without the need for transformation, as previously required for VNC_conv_-based calcium assessment [6, 7, 9, 19, 20]. Moving organs are always a challenge in medical imaging, because they are prone to artifacts. The same is true for cardiac imaging, which is why beta-blockers are often used to reduce heart rate [21]. However, there are patients, with contraindications to the use of beta-blockers who must be scanned at higher heart rates [22].

In their phantom study, Werf et al. found heart rate-induced variations in CAC extent measured on TNC for both high-end energy-integrating [10] and photon-counting detector [23] CT systems. Higher heart rates decrease the reproducibility of CAC measurements, as found in phantom [24] and in patient cohort [25] studies. In this study, no significant heart rate-related differences were found for either VNC_conv_- or VNC_pc_-based CAC quantities or global noise levels, when considering only intra-reconstruction comparisons (Figs. 2a and 4a). These results are consistent with Brodoefel et al., [26] who found heart rate-independent image quality in dual-source CTA compared to invasive angiography, but instead found a correlation with heart rate variability and calcification extent. Another study concluded that dual-source CT angiography provides high diagnostic accuracy independent of the heart rate [27]. However, this study compared higher and lower heart rates according to a single threshold.

Focusing on differences between reconstructions, the reproducibility of VNC-based CAC scores was found to be much lower compared to ground truth with a higher variability in measured quantities. Again, it should be noted that the apparently smaller interquartile ranges of VNC_conv_ would naturally increase to a range similar to that of VNC_pc_ if the transformation were applied. The percentage deviation from the reference TNC values increased with rising heart rate. Given a greater calcification burden than simulated with the phantom insert, this discrepancy may increase accordingly, leading to misclassification in risk categorization.

Noise, on the other hand, was significantly reduced on VNC reconstructions compared to TNC. Jungblut et al. measured noise in the lung parenchyma using a technique similar to that used in this study and compared TNC to VNC_conv_ both derived from PCD-CT, without reporting significant or large differences [28]. These discrepant results may be due to reconstruction with a sharp lung kernel compared to the soft tissue kernel used in this study.

This study has several limitations. First, the ad hoc phantom was created manually, so there is a possibility that the differences between TNC and VNC are not entirely algorithmic. Second, the comparisons are based on small sample sizes (9 vs. 9 measurements each), which limits their power, especially for tests of significance. Third, ideal heart movements were simulated without taking into account heart rate variations or arrhythmias. Fourth, all evaluations are entirely objective and based on measurements. Further studies should assess the subjective image perception.

In conclusion, VNC-based calcium quantification and noise assessment showed no dependence on heart rate in an intra-reconstruction comparison. Although the difference between the calcium-sensitive VNC_pc_ algorithm and the ground truth was not significant in the assessment of lesion number, Agatston score, and calcium volume, the average deviation increased with higher heartrates in the mean. The high variability of measured CACS on VNC reconstructions indicates poor reproducibility and holds the potential for incorrect risk classification.

### Supplementary Information

Below is the link to the electronic supplementary material.

Demonstration of the performed global noise measurements. a shows the three selected slices within the heart volume and b their respective noise maps. The rectangular boarder marked in red differentiates between the moving heart cylinder and the static background of the phantom. In c for both regions the histograms are plotted which most frequent standard deviation in HU was taken as global noise measure.
Supplementary file1 (TIF 10625 kb)

## Abbreviations

CAC: Coronary artery calcium
CT: Computed tomography
CTDI_vol_: Volumetric CT dose index
DLP: Dose length product
PCD: Photon-counting detector
ROI: Region of interest
SSDE: Size-specific dose estimate
TNC: True non-contrast
VNC: Virtual non-contrast (conv = conventional, pc = PureCalcium)
