# Supplementary figures and images for: Heart rate sensitivity of virtual non-contrast calcium scores derived from photon counting detector CT data: a phantom study
Source: Radiol Med. 2024 Feb 6;129(3):401–10. doi: 10.1007/s11547-024-01773-3 (PMC10943147; doi:10.1007/s11547-024-01773-3)

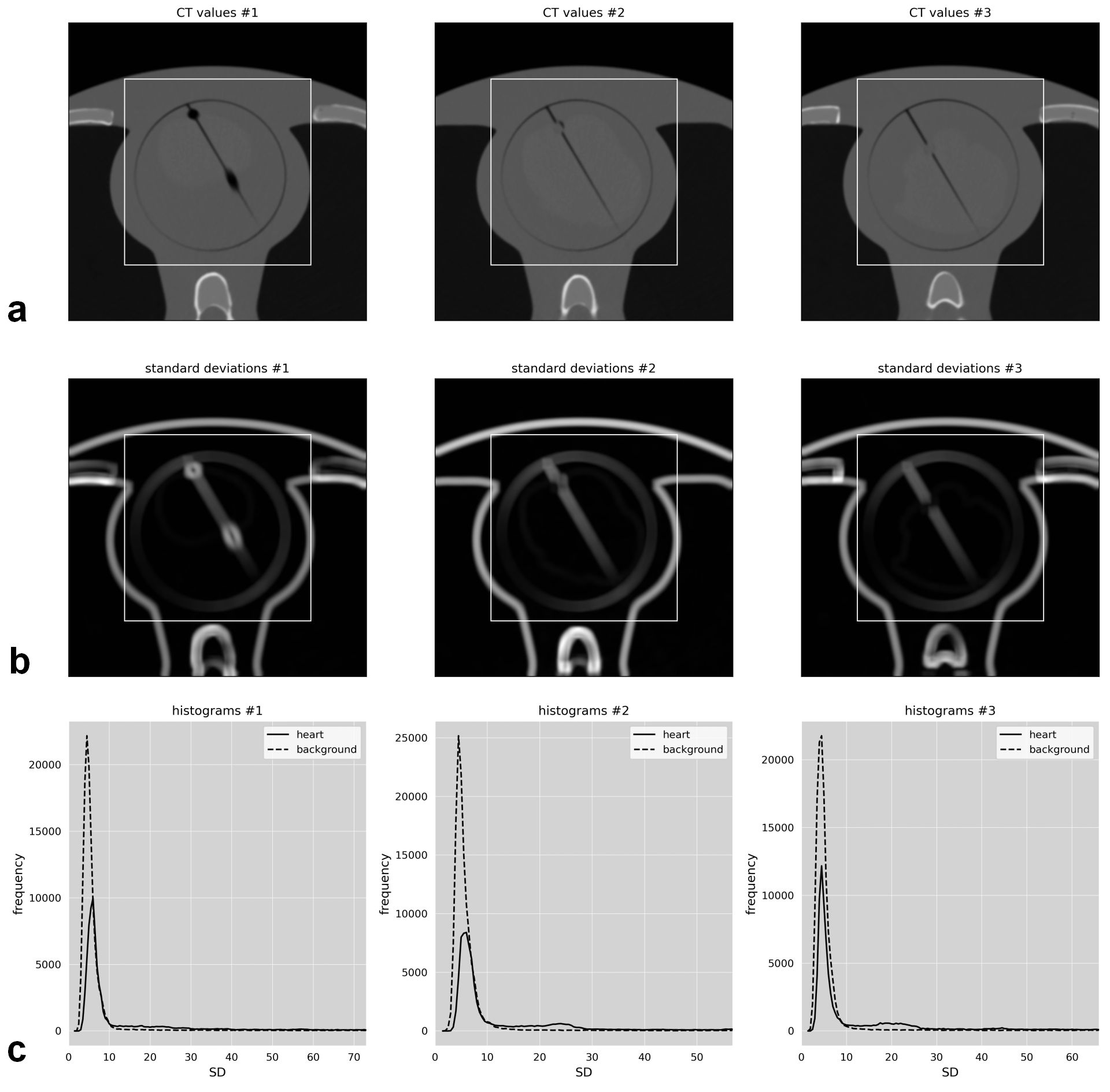

Supplement: Supplementary file 1 — Demonstration of the performed global noise measurements. a shows the three selected slices within the heart volume and b their respective noise maps. The rectangular boarder marked in red differentiates between the moving heart cylinder and the static background of the phantom. In c for both regions the histograms are plotted which most frequent standard deviation in HU was taken as global noise measure. Supplementary file1 (TIF 10625 kb) [file 11547_2024_1773_MOESM1_ESM.tif]
